# Short microsecond pulses achieve homogeneous electroporation of elongated biological cells irrespective of their orientation in electric field

**DOI:** 10.1038/s41598-020-65830-3

**Published:** 2020-06-04

**Authors:** Janja Dermol-Černe, Tina Batista Napotnik, Matej Reberšek, Damijan Miklavčič

**Affiliations:** University of Ljubljana, Faculty of Electrical Engineering, Tržaška cesta 25, 1000 Ljubljana, Slovenia

**Keywords:** Cell biology, Cardiology

## Abstract

In gene electrotransfer and cardiac ablation with irreversible electroporation, treated muscle cells are typically of elongated shape and their orientation may vary. Orientation of cells in electric field has been reported to affect electroporation, and hence electrodes placement and pulse parameters choice in treatments for achieving homogeneous effect in tissue is important. We investigated how cell orientation influences electroporation with respect to different pulse durations (ns to ms range), both experimentally and numerically. Experimentally detected electroporation (evaluated separately for cells parallel and perpendicular to electric field) via Ca^2+^ uptake in H9c2 and AC16 cardiomyocytes was numerically modeled using the asymptotic pore equation. Results showed that cell orientation affects electroporation extent: using short, nanosecond pulses, cells perpendicular to electric field are significantly more electroporated than parallel (up to 100-times more pores formed), and with long, millisecond pulses, cells parallel to electric field are more electroporated than perpendicular (up to 1000-times more pores formed). In the range of a few microseconds, cells of both orientations were electroporated to the same extent. Using pulses of a few microseconds lends itself as a new possible strategy in achieving homogeneous electroporation in tissue with elongated cells of different orientation (e.g. electroporation-based cardiac ablation).

## Introduction

When short, high voltage pulses are applied to biological membranes, cell electroporation occurs^1^. Cell membrane permeability increases, presumably due to pore formation^2^. If cells recover after electroporation, it is considered as reversible electroporation. If the cells do not recover and they die, it is considered as irreversible electroporation. Electroporation is used in medicine^3^ (electrochemotherapy^4–6^, gene electrotransfer^7,8^, transdermal drug delivery^9^, irreversible electroporation as a method of tumor tissue ablation^10,11^, electrophysiology/cardiac ablation^12–14^), food technology^15,16^, and biotechnology^17,18^.

Two clinically relevant treatments with electroporation are gene electrotransfer of skeletal muscles^19,20^, for example for DNA vaccination^21^ against HIV, human papillomaviruses, influenza^22–25^, and cardiac ablation of cardiomyocytes in treatment of heart arrhythmias^26–30^. In these treatments, electric pulses are applied to muscle cells, which are typically elongated and oriented. It was shown experimentally *in vitro* and *in vivo* as well as numerically that with eight 100 µs long pulses, electroporation threshold depends on muscle orientation^31,32^. The electric field of the same strength parallel to the skeletal muscle fibers caused more dye uptake and accordingly, a greater extent of electroporation of cells than when applied perpendicularly. The same observation was found when cardiomyocytes were exposed to electric pulses of ms range (from 2 ms to 20 ms)^33–35^. Due to the elongated shape of muscle cells and variable orientation of muscle fibers in tissue, homogeneous electroporation with longer pulses is thus difficult to achieve. We can either electroporate only a part of the tissue by applying a low electric field or damage the tissue by applying a high electric field. Not just muscle cells, with electroporation, we treat different tissues consisting of cells of various shapes, sizes, and orientations. Also in preclinical studies *in vitro*, attached cells in cultures are usually differently shaped and oriented^36^.

In studies with shorter pulse durations (1 µs and shorter), it is often claimed that shorter pulses affect tissue more homogeneously than longer pulses. Lately, pulse durations have decreased down to nanoseconds^37^, and picoseconds^38^ and different pulse shapes are getting more popular (biphasic^39,40^, sinusoidal^41^). Higher frequency spectral content of the short high-frequency biphasic (bipolar) pulses was also suggested to reduce the inhomogeneity of tissue impedance^42^. It was suggested that because plasma membrane cannot get fully charged when pulses shorter than cell membrane time constant, which is in the range of 1 µs, are applied, the cell dimension in the direction of the electric field had little or no impact on the threshold transmembrane voltage for electroporation^43,44^. Another consequence of incomplete cell membrane charging is that nanosecond pulses also influence intracellular organelles^37,45,46^.

In our study, we aimed to determine how cell orientation influences electroporation with respect to the electric field in a wide range of pulse durations. Our hypothesis was that cell orientation influences electroporation with single electric pulses of different pulse durations differently. We addressed the problem from experimental as well as modeling point of view for pulse durations of 100 ns to 10 ms in experiments and 10 ns to 100 ms in modeling. We focused on one pulse parameter – pulse duration, therefore, we used simple, single monopolar pulses. Namely, effect of several consecutive pulses on cell membrane electroporation is still under debate with effect as cell sensitization^47^ and cancellation effect^48^ being reported and debated. Also, the effect of several pulses on pore formation is not adequately described with the existing asymptotic pore equation and thus applying the asymptotic pore equation to several pulses in the modeling study would be questionable. Specifically, we focused on cardiomyocytes, which are the target cells in cardiac ablation.

Surprisingly, we observed that at pulse durations in the nanosecond range, cells oriented perpendicular to the electric field were more electroporated than cells oriented parallel to the electric field. Cell orientation had an almost negligible effect on cell electroporation when pulses were in the range of a few microseconds. However, in agreement with existing knowledge, we observed that at longer pulse durations, cell orientation parallel to the electric field was more efficient than perpendicular. With these results, we have proven our hypothesis that cell orientation influences electroporation with single electric pulses of different pulse durations. However, the exact mechanism for this phenomenon is still to be elucidated.

To achieve more homogeneous and orientation independent electroporation of muscle cells (or other elongated cells), we propose an additional protocol besides the already existing way of delivering pulses in different directions^49,50^. Namely, we can deliver pulses of duration in the range of a few microseconds where cell orientation has the least effect on electroporation. Also, with a few microsecond long pulses, we could avoid or at least minimize muscle contractions and pain as recently suggested^51,52^. Thus, more homogeneous and cell orientation independent electroporation could be achieved.

## Results

### Experiments

**Figure 1:**
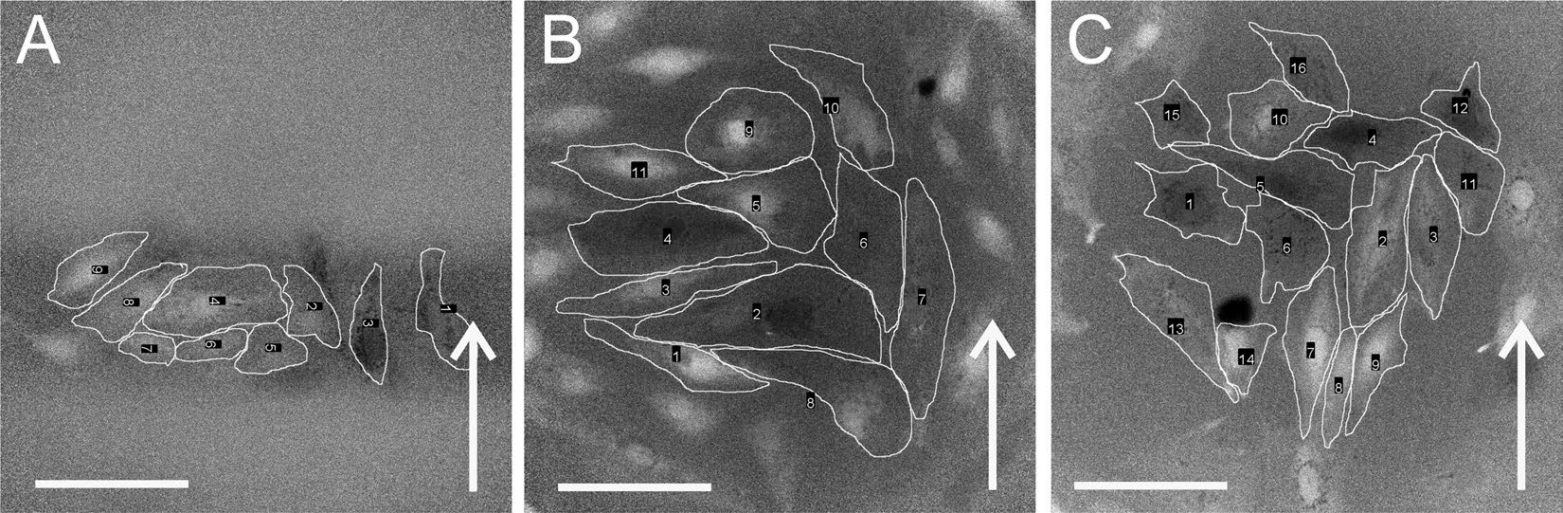
Electroporation of H9c2 cells as monitored by Ca^2+^ uptake with fluorescent calcium indicator Fura-2 (ratio images). Cells were pulsed with single pulses of (**A**) 100 ns, 40 kV/cm, (**B**) 10 µs, 1000 V/cm, (**C**) 1 ms, 200 V/cm. Cells at 8 s after pulse application are encircled, brighter cells express higher levels of Ca^2+^ uptake. Scalebar: 100 µm. Arrow: the direction of the electric field.

H9c2 rat cardiac myoblasts and AC16 human cardiomyocytes were subjected to a single pulse of 100 ns of different electric field strengths (for electric field estimation, see Supplementary Fig. S1). For electric pulses of 1 µs to 10 ms duration, they were subjected to consecutive electric pulses of increasing electric field strength (approximated as a ratio of voltage to distance between the inner edges)^39^ and a pause for recovery in-between (8 to 12 min, depending on the applied voltage, to restore low internal calcium concentration). Successful electroporation was determined by internal calcium concentration increase due to the uptake of calcium ions from the surrounding medium (Fig. 1; for the method see Methods, Exposure of cells to electric pulses, and Supplementary Fig. S2) as detected with an increased Fura-2 340/380 ratio under a fluorescence microscope. A higher Fura-2 340/380 ratio is a consequence of a higher calcium concentration. The peak calcium concentration was in most cases detected 8 s after pulse application (applied between second and third acquired image). The ratio over time graph of a typical Fura-2 signal is given in the Supplementary Fig. S3. Intracellular calcium concentration increased with increasing electric field strength.

**Figure 2:**
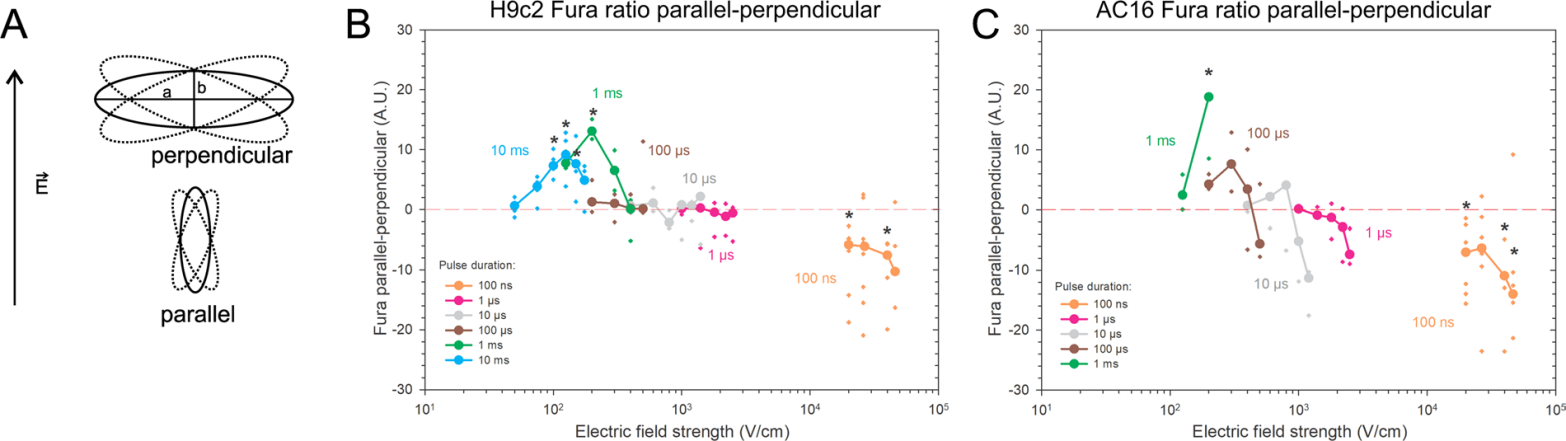
Experimental results of electroporation of parallel and perpendicular cardiomyocytes H9c2 and AC16, using pulses of different durations (100 ns to 10 ms) at different electric field strength. (**A**) Parallel and perpendicular cells are elongated (a > 2b), and their longer axes (a) are oriented parallel or perpendicular to the electric field ***E***, with 20° tolerance in angle. (**B**), (**C**) Experimental electroporation of cells of different orientations was monitored by calcium uptake with a fluorescent calcium indicator Fura-2, 8 s after the pulse application. Results are expressed as a median difference in Fura-2 340/380 ratio between parallel and perpendicular cells (lines), and individual measurements are shown for each electric field strength (crosses). When parallel cells are more affected than perpendicular, then the difference is a positive value. When perpendicular cells are more affected, then the results are below zero. B) Experimental results of H9c2 cells were obtained from 5–30 cells per experiment, an average of three independent experiments, except for 1 ms (repeated 4×), 10 ms (repeated 5×), 100 ns, 40 and 46.6 kV/cm (repeated 5×), 100 ns, 20 kV/cm (repeated 6×), and 100 ns, 26.6 kV/cm (repeated 9×). C) Experimental results of AC16 cells were obtained from 4–23 cells per experiment, an average of three independent experiments, except for 100 ns, 40 kV/cm (repeated 5×), 100 ns, 46.6 kV/cm (repeated 6×), 100 ns, 20 kV/cm (repeated 7×) and 100 ns, 26.6 kV/cm (repeated 7×). * - statistically significant differences from control (p < 0.05), the Kruskal-Wallis One Way Analysis of Variance on Ranks, followed by Multiple Comparisons versus Control Group (the Dunn’s Method). Results of statistical analyses are shown in the Appendix, Tables A1a and A1b.

Most of the cells of both cell lines were elongated (Fig. 1 and Supplementary Fig. S4), with their long axis at least twice the size of their short one (a > 2b, see Fig. 2A). The cells were analyzed with respect to their orientation in the electric field (Fig. 2A) and the difference in Fura-2 340/380 ratio between cells parallel and perpendicular to the electric field was determined (Fig. 2).

When cells were exposed to short, 100 ns pulses, cells oriented perpendicular with respect to the electric field were significantly more affected than cells oriented parallel with respect to the electric field (Figs. 1A, 2B,C). Therefore, the difference in Fura-2 340/380 ratio parallel-perpendicular was below zero. When H9c2 were exposed to a single 100 ns pulse with an electric field of 40 kV/cm (Fig. 2B), cells perpendicular to the electric field exhibited higher calcium uptake than cells parallel to the electric field (the median difference in Fura-2 340/380 ratio parallel-perpendicular was −7.58 arbitrary units A.U., compared to non-pulsed control cells with the median difference of 0.09 A.U.). Similarly, perpendicular AC16 cells exposed to 100 ns, 40 kV/cm pulses showed higher calcium uptake than parallel cells (see Fig. 2C, the mean difference in Fura-2 340/380 ratio parallel-perpendicular was −10.93 A.U., compared to non-pulsed control cells with the mean difference of −0.08 A.U.).

When cells were exposed to single pulses of intermediate durations of 1 µs to 100 µs (Figs. 1B, 2B,C), the difference in Fura-2 340/380 ratio in cells parallel and perpendicular to the electric field was close to zero and not significantly different from control where the difference was around zero. In this range, the cells of both orientations, parallel and perpendicular, were electroporated to a similar extent.

With longer, 1 and 10 ms pulses, however, we observed significantly higher calcium uptake in parallel cells than in perpendicular (Figs. 1C, 2B,C); therefore, the Fura-2 340/380 ratio difference between parallel and perpendicular was above zero. The median difference in Fura-2 340/380 ratio between parallel and perpendicular H9c2 cells after applying a single 1 ms, 200 V/cm electric pulse was 13.07 A.U. (Fig. 2B). In non-pulsed control in experiments, using 1 ms pulse, the median difference between parallel and perpendicular cells was −0.01 A.U. Similarly, the median difference in Fura-2 340/380 ratio between parallel and perpendicular AC16 cells after applying a single 1 ms, 200 V/cm electric pulse was 18.81 A.U. (Fig. 2C), whereas the ratio difference in non-pulsed control cells in experiments using 1 ms was 0.01 A.U.

The crossover of more affected cells from perpendicular to parallel, therefore, lies in the pulse duration range between 1 and 100 µs. The crossover for AC16 may occur at longer pulse durations than for H9c2 since, at 10 µs pulse duration, the median difference is still well below zero although not significantly different from control (−11.31 A.U. in cells exposed to a single 10 µs pulse, 1200 V/cm, and −0.01 A.U. in non-pulsed control cells, see Fig. 2C). On the other hand, the differences between parallel and perpendicular cells might be more pronounced in AC16 than in H9c2.

### Modeling

When one pulse of durations from 10 ns to 100 ms was applied either in parallel or perpendicular to the cells’ long axis, the pore density increased. By integrating the pore density, we determined the number of pores formed on the cell membrane.

**Figure 3:**
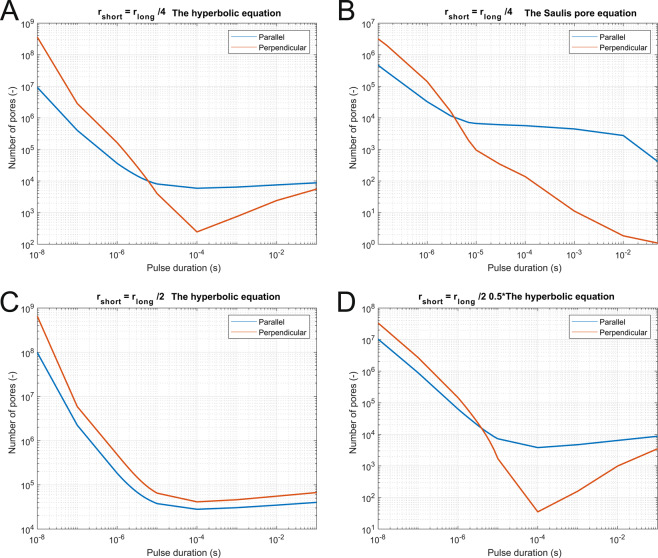
Numerically determined number of pores formed on the cell membrane as a function of pulse duration when pulses of equivalent parameters are applied to two different cell geometries. (**A**) Short axis was one-quarter of the length of the long axis, and equivalent parameters were obtained with the hyperbolic equation (crossover at 6 µs), or (**B**) the Saulis pore equation (crossover at 3 µs). (**C**) Short axis was one half of the length of the long axis, and equivalent parameters were obtained with the hyperbolic equation (no crossover observed) or (**D**) the hyperbolic equation, scaled for a factor of 0.5 to take into account larger cell geometry (crossover at 4 µs).

In Fig. 3, we can observe the number of pores formed when an electric field is applied either perpendicular or parallel to the cells’ long axis for two geometries of cells (geometry ratio 1:4 on A and B and ratio 1:2 on C and D). The equivalent pulse parameters were determined using two different equations – the hyperbolic equation and the Saulis pore formation equation. In three of the graphs shown (A, B, D), we can see that at shorter pulses, cells with perpendicular orientation were more affected, while at longer pulse durations, the cells with parallel orientation were more affected. This crossover from cells with perpendicular orientation to cells with parallel orientation being more affected occurred between 3 µs and 5 µs in all cases (Fig. 3A,B,D) and could be thus observed at 1:2 as well as 1:4 ratios of the lengths of the long and short axis of the elongated spheroid. Figure 3C shows that if a higher electric field (twice-higher electric field than in Fig. 3D) is applied to a larger cell (compare sizes on Fig. 7A,B), more pores form than in other three cases, most of the cell membrane is electroporated, both orientations of the electric field become equivalent and the crossover is not observed anymore. For each pulse duration, also the applied electric field is important regarding the efficiency of electroporation of parallel vs perpendicular orientation (Supplementary Fig. S5). With 100 ns pulses, the perpendicular orientation was always more efficient, while for longer pulses it depended on the applied electric field.

**Figure 4:**
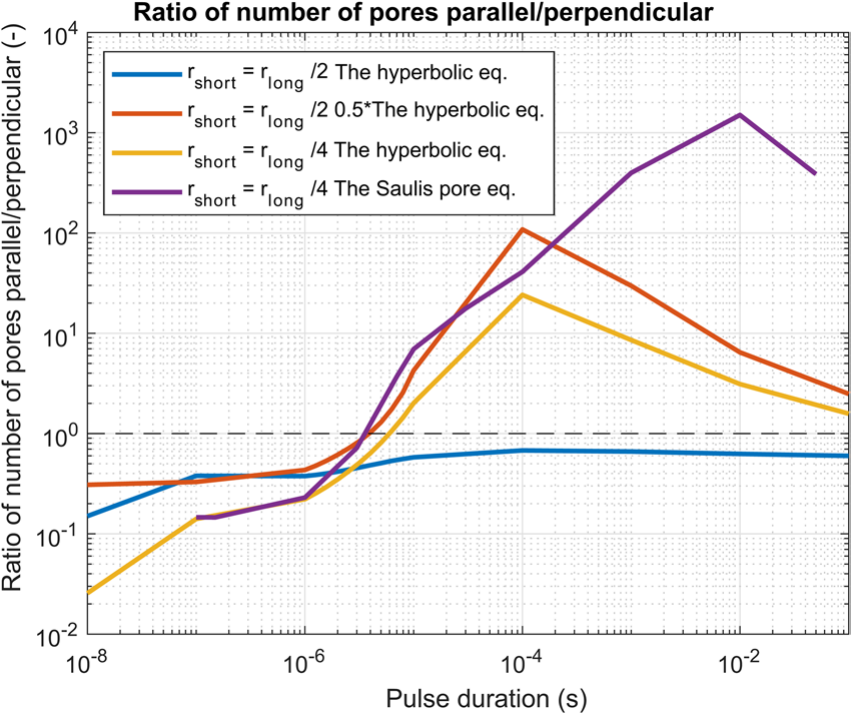
A numerically determined ratio of the number of pores when cells orientation is parallel vs perpendicular. Different examples are shown: when a cell is of different geometry (ratio 1:2 and 1:4) and/or when equivalent pulse parameters are calculated as the hyperbolic equation, scaled hyperbolic equation or with the Saulis pore equation. We can see that in all cases with nanosecond pulses the perpendicular direction was more efficient in pore formation and the crossover was obtained in the range of 3 to 6 microseconds, depending on the cell geometry and applied an electric field. The black dashed line shows the ratio of 1 where perpendicular and parallel orientation are equivalent.

**Figure 5:**
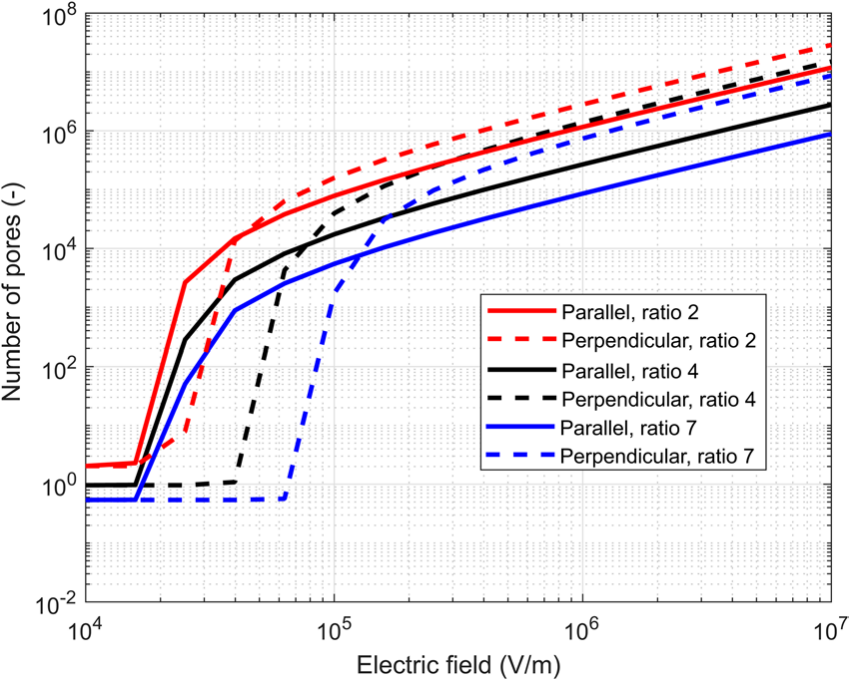
Number of pores formed as a function of the applied electric field when a single 100 µs long pulse is applied. The effect of different ratios of geometry is shown for the geometry ratios 2 (in red), 4 (in black) and 7 (in blue). For all, parallel orientation is in solid line and perpendicular in dashed. For all ratios and parallel orientation, the pores start forming at approximately the same electric field, while for perpendicular orientation, a higher electric field is needed for more elongated cells (ratio 7) than for less elongated cells (ratio 2) to achieve a similar level of electroporation.

**Figure 6:**
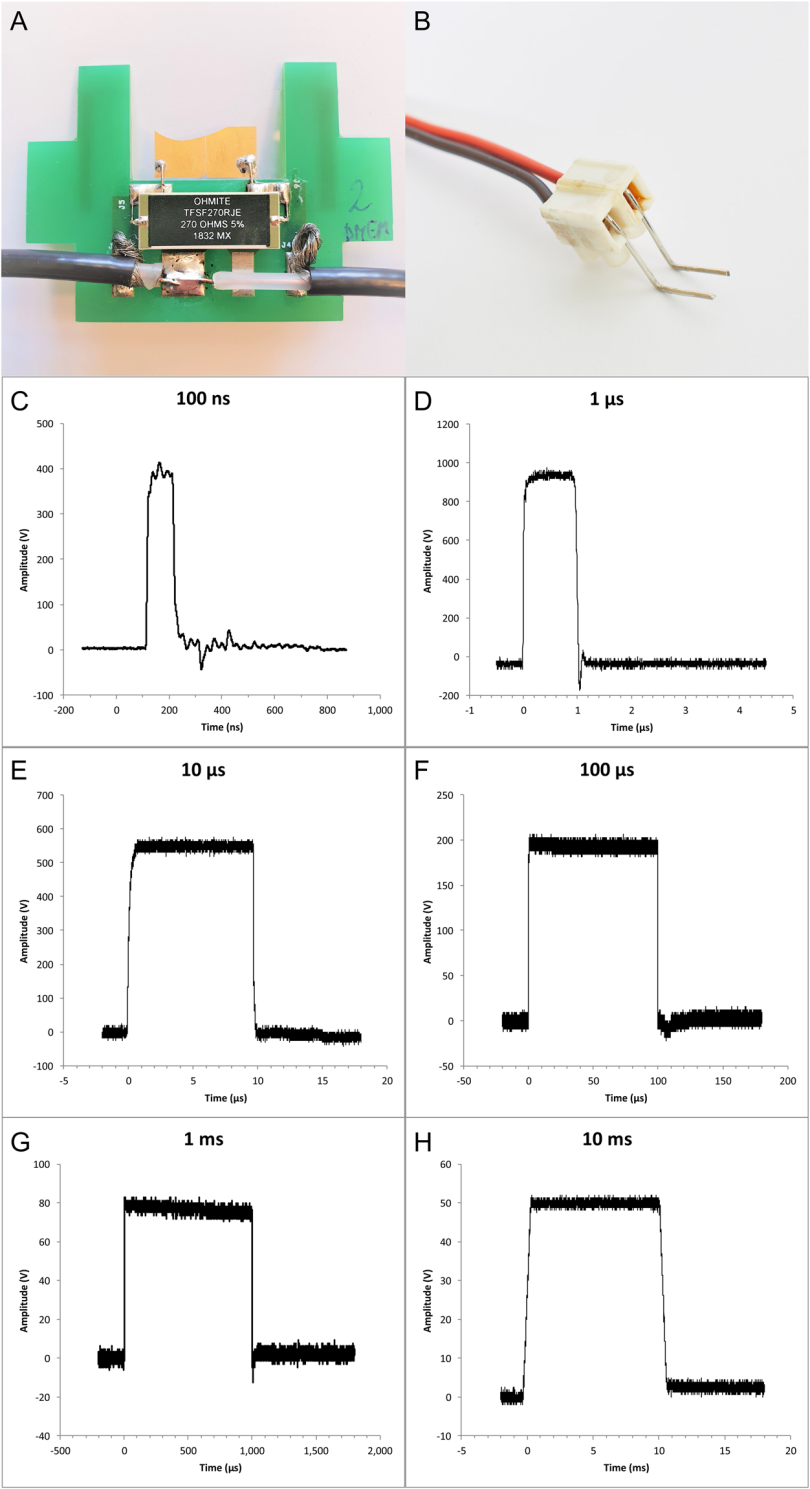
Electrodes and waveforms of pulses used in the study. Electrodes for (**A**) 100 ns pulse application, and (**B**) for 1 µs – 10 ms pulse application. Waveforms of pulses used in the study: (**C**) 100 ns, 400 V (numerically calculated electric field of 26.6 kV/cm), (**D**) 1 µs, 1000 V (voltage-to-distance ratio 2500 V/cm), (**E**) 10 µs, 600 V (voltage-to-distance ratio 1500 V/cm), (**F**) 100 µs, 200 V (voltage-to-distance ratio 500 V/cm), (**G**) 1 ms, 80 V (voltage-to-distance ratio 200 V/cm), (**H**) 10 ms, 50 V (voltage-to-distance ratio 125 V/cm).

**Figure 7:**
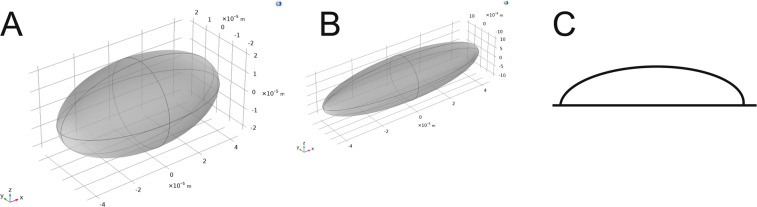
Geometry of the modeled spheroids. In the model, we modeled different spheroid geometries. (**A**) The modeled geometry of the cell with ratio 2× and (**B**) with the ratio 4×. (**C**) In experiments, cells were attached to the bottom of the dish and shaped like half of an elongated spheroid. However, due to the numerical symmetry of the problem, we modeled the cells as full spheroids.

Figure 4 summarizes the results of Fig. 3 as we show the ratio of pore number of the parallel to the perpendicular orientation. This ratio is below one in the nanosecond range (where cells with perpendicular orientation are more affected), is around one in the range of a few microseconds (cells with perpendicular and parallel orientation are similarly affected), and is much higher than one in the millisecond range (cells with perpendicular orientation are less affected). Similar results were obtained if we considered the electroporation to be a threshold phenomena when more than 10^13^ pores/m^2^ are formed on the membrane (Supplement, Fig. S7).

In Fig. 5, the effect of cell geometry is more evident. The number of formed pores is shown for three different cell geometries (ratio long axis to short axis 2, 4 or 7). We can see that for all ratios, i.e., cell shapes, in a parallel orientation, pores start forming at approximately the same electric field. On the contrary, for perpendicular orientation, much higher electric fields are needed for the more elongated cells than for less elongated cells to achieve the same effect. To see the difference more clearly, we can divide the number of formed pores for parallel vs perpendicular orientation, obtain only one curve for each geometry ratio, and we can see that the ratio is above 1 for lower electric fields and below 1 for higher electric fields (Supplement, Fig. S6).

## Discussion

### Aims and general observations

In DNA vaccination of muscles as well as in cardiac ablation by irreversible electroporation, differently oriented muscle fibers may represent an obstacle in achieving homogeneous electroporation. We thus studied how cell orientation influences electroporation with respect to the direction of electric field in a wide range of pulse durations, conducting experimental as well as modeling study. We observed that nanosecond pulses caused more pronounced electroporation of the cells with perpendicular orientation to the electric field than the cells with parallel orientation. In a range of a few microsecond long pulses, a crossover was observed where cell orientation was not influencing electroporation efficiency. With longer micro- to millisecond pulses, the cells with parallel orientation were electroporated more than cells with perpendicular orientation to the electric field.

Our study is important for clinical use of electroporation of elongated spheroidal cells, for example, muscle cells in tissues targeted with DNA transfer for DNA vaccination and gene therapy and cardiomyocytes for cardiac ablation. To achieve pulmonary vein isolation with irreversible electroporation also termed pulsed field ablation, a transmural ablation, penetrating the wall of the heart circumferentially around the vein, has to be achieved. The necessity for the transmural and continuous ablation means treating cells of significantly different orientations with respect to the direction of electric field and thus, achieving a uniform, and cell orientation independent electroporation could assure reliable treatment efficiency.

### Methodology

We evaluated the effect of cell orientation in a wide range of pulse durations experimentally *in vitro* by measuring calcium influx into cardiomyocytes of elongated spheroidal shape (Table 1, results on Fig. 2B,C) as well as numerically by calculating the number of formed pores as well as membrane area electroporated (Figs. 3–5). Numerical analysis was necessary as analytical was not possible due to the non-uniform thickness of the cell membrane at the poles and the equator^53^ and the asymptotic pore equation not being calculable analytically. The comparison of experimentally determined intracellular calcium concentration and numerically determined number of pores formed was possible as we assumed that the transport of calcium occurs through pores or at least that the transport of calcium is proportional to the number of pores. Thus, the number of pores formed is proportional to intracellular calcium concentration.

**Table 1:** Applied electric field strengths at different pulse durations used in experiments.

Pulse duration	Applied electric field strengths
100 ns	20, 26.6, 40, 46.6 kV/cm
1 µs	1000, 1400, 1800, 2200, 2500 V/cm
10 µs	400, 600, 800, 1000, 1200, 1400 V/cm
100 µs	200, 300, 400, 500 V/cm
1 ms	125, 200, 300, 400 V/cm
10 ms	50, 75, 100, 125, 150, 175 V/cm

**Figure 8:**
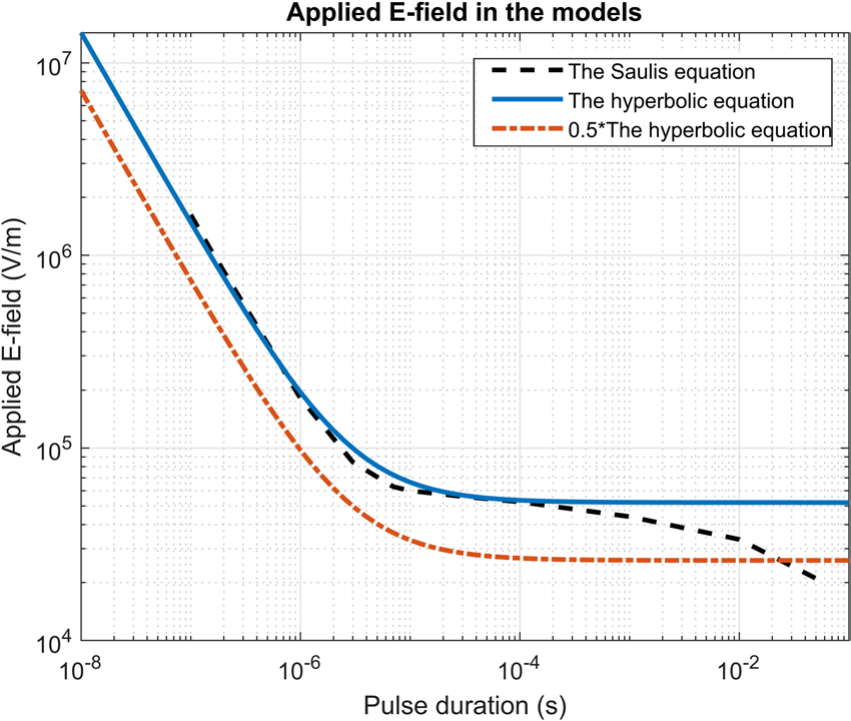
Strength-duration curves which were used to scale the electric field to obtain equivalent pulse parameters. Black dotted line was obtained by optimizing the Saulis pore equation, the solid blue line is the hyperbolic equation, and the orange dash-dot line is the hyperbolic equation decreased for a factor of 0.5^54^. Factor 0.5 was chosen to compensate for a smaller cell in calculations with ratio 4×.

In the numerical part of our study, the applied electric field was adapted for different pulse durations to obtain an iso-effect, i.e. similar pore density^52,54^ (Fig. 8). Similarly, we applied a higher electric field with shorter pulses than with longer pulses to obtain a similar calcium influx (up to 46.6 kV/cm for 100 ns pulses and 175 V/cm for 10 ms pulses, see Table 1) in the experimental part of our study. More precisely, electroporation depends, among others, on the induced transmembrane voltage. Based on the Schwan equation, isolated larger spherical cells under physiological conditions are electroporated at lower electric fields than smaller cells^55^, although some experimental results show different results, presumably due to different detection methods^56^. Thus, we adapted the numerically applied electric field when the ratio of the spheroid’s axes was changed (1:4 and 1:2) as with ratio 1:2 cells were larger due to larger semi-axis b (Fig. 7A,B). At higher electric fields, cell orientation became less important experimentally (Fig. 2B,C) as well as numerically (Fig. 3C). With higher electric fields, most of the cell membrane has increased pore density. The induced transmembrane voltage (ITV) first increases and then due to pore formation flattens where a critical value is exceeded (on the poles of the cell)^57,58^. With increasing electric field, increased ITV progresses to the equator of the cells. If electric field is high enough, the high ITV comes to the equator and majority of the cell membrane is thus electroporated, making cell orientation a marginal influence. In experiments, saturation of the dye also plays an important role in detecting calcium uptake at higher electric fields. Thus, different cell orientations are not critical close to the electrodes but become critically important where the electric field drops, e.g., at the margin of the treatment zone.

In our modeling study, we had to resort to certain approximations. Namely, as the pulse duration decreases, the frequency content of the pulse is increased. The properties of biological cells are frequency-dependent^59^, and in general, at frequencies higher than 1 MHz, the conductivity of cells/tissues starts to decrease and the permittivity to increase. In our study, we assumed the properties of the cell membrane and cytosol to be frequency independent. A possible upgrade is modeling dielectric properties as frequency-dependent, which is particularly relevant when short nanosecond pulses are applied^60^. Also, we described pore formation by the non-linear asymptotic equation, which assumes that only the density of the pores changes while their radii remain the same. Possible improvement of the model is including pore growth which becomes increasingly more important when longer pulses are applied^61^. Size of the cells in the model was adapted to the sizes of cells in the experiments. From images under the microscope, we could obtain information on the size in two dimensions (semi-axes a and b). However, we did not have information on the size in the third dimension (height, z-axis). As the bottom of the cell dish is electrically insulated, the numerical problem was symmetrical, and results were the same if a whole cell (Fig. 7A,B) or only one half (Fig. 7C) was modelled. Additionally, the assumed symmetry of cells in *x*- and *y*-axis enabled us to model only 1/8 of the cell, which renders calculations faster. The results of real shaped cells as obtained from e.g. a z-stack using confocal microscopy could be also considered as important but as it was shown recently, the modeling of (primary) cardiomyocytes as prolate spheroids is a good approximation approximation of stationary state with decreased computational cost in comparison to modeling of the real 3D geometry^62^.

### From nanosecond to millisecond pulses

Our results at shorter pulses are in agreement with^63^ where it was numerically shown that pulses in the nanosecond range are more efficient when delivered perpendicular to the long axis than parallel presumably due to flattening of the cell surface on the pole of the cell, although a definite reason was not found. On the contrary, it was observed experimentally that cell shape, size, and orientation did not have influence with 600 ns long pulses^64^, although the cells used in that study were not as elongated as in our study. However, with 600 ns long pulses, we are already approaching the microsecond pulses.

Contrary to our results, it was previously shown that cell orientation did not affect the electroporation threshold for 200 or 800 ns pulses^43^. Still, a two-fold reduction in the threshold of electroporation was observed for longer pulses of 50 µs and 200 µs, when cell orientation was changed from perpendicular to parallel, which is consistent with our results. On Fig. 1c in the paper^43^, we can see that there is an indication of the crossover (i.e., change from perpendicular to parallel orientation being more efficient) at around 2 µs, consistent with our data (modeled crossover between 3 and 6 µs and measured between 1 and 100 µs). There are two main differences between the studies which could explain the discrepancy in observations. 1) We tested shorter 100 ns pulses, and it is possible that they did not observe this crossover as the whole range of pulse durations was not investigated but they only compared long to short pulses. 2) We worked with cell culture of ventricular myocytes as opposed to primary ventricular myocytes which have a distinct square shape and a distinct network of T-tubules that could affect the distribution of the induced transmembrane voltage and electroporation, especially in the nanosecond range.

It seems that the optimal pulse duration for treating tissues with different cell orientations is in the range of 1–10 µs where the orientation has a negligible effect on electroporation extent. This range may vary with cell type, size, shape, and electric field, which is in agreement with^43^. In our experiments, the effect of pulses on cells was slightly different in the two cells lines. Namely, AC16 became rounded at higher electric fields, similar to isolated rabbit cardiomyocytes^34^; however, in H9c2 cells, this phenomenon was not observed. Also, the crossover of more affected cells from perpendicular to parallel may occur at longer pulse durations in AC16 than in H9c2 since, at 10 µs duration, the difference in ratio between parallel and perpendicular AC16 cells was still well below zero although not significantly different from control which is close to zero (Fig. 2C).

With longer pulses, our results show that cell shape and orientation have a large effect on electroporation outcome with parallel orientation being more efficient than perpendicular. In previous reports in agreement with our results, parallel orientation being more efficient than perpendicular was observed *in vitro* on cell lines and primary cells (cardiomyocytes), *in vivo* and in simulations^31,33–35,65^.

In the case of longer micro- and millisecond pulses, the maximum change in membrane potential occurs at the ends that are close to the electrodes. Threshold of the electroporation is proportional to the cell length in the direction of the field. Therefore, perpendicular cells have higher permeabilization threshold than parallel cells^34,35^ i.e. require higher pulse amplitudes. Moreover, higher membrane curvature facilitates electroporation^36^, but also surface tension may play an important role^66^. In the case of nanosecond pulses, additional mechanisms must be responsible for this observed opposite dependency with perpendicular orientation being more efficient than parallel. The main difference between the two is the pulse duration which influences the pore formation in a different fashion, probably due to charging of the membrane (Maxwell-Wagner polarization for longer pulses vs dielectric stacking for nanosecond pulses)^43^ and curvature of the membrane^63^. However, the exact mechanism remains to be elucidated which is a subject of our future research.

### Cell lines vs primary cardiomyocytes

Our experiments were performed *in vitro*, where we tested many different pulse lengths without ethical considerations and restrictions and where cell geometry and orientation can be easily determined. The two cell lines used in our study, H9c2 and AC16 are of cardiac origin (rat ventricular myoblasts and human ventricular myocytes, respectively). Both cell lines show many morphological and physiological characteristics typical for cardiac cells; however, they also differ from primary cardiomyocytes and especially, from the cells in *in vivo* conditions. Cells of both cell lines are elongated, spindle-shaped, and growing in parallel arrays and resemble primary cardiomyocytes; however, the length to width aspect ratio here is much lower than in primary cardiac cells (where around 7^43,67^), and the structure of the plasma membrane of cardiomyocytes is very different on the ends vs sides^67^. H9c2 show some characteristics of immature embryonic cardiomyocytes and skeletal muscle cells but have preserved surface coat and several electric and metabolic processes found in adult cardiac cells^68–70^. AC16 show more structural and biochemical characteristics of adult cardiac cells, however, are also in precontractile stage and lack action potential^71^. Different morphological and physiological characteristics between cardiac cells *in vivo* and cell lines used in our study could thus lead to different results in *in vivo* conditions as in our *in vitro* study. Therefore, our results may not be directly translated to *in vivo* situation.

We addressed the question of different elongation of cell lines vs primary cardiac cells in by modeling pore formation on cells of three different cell geometries (Fig. 5) – with the ratio long to the short axis of 2, 4 or 7. In our experiments, the ratio was around 3.5 for both cell lines, while for primary cells it is around 7. We observed that the non-uniform electroporation of differently oriented cells is more pronounced with more elongated cells than with less elongated cells. Thus, we assume that with primary cells, the effect would be even more pronounced than observed in our cell lines. Unfortunately, studies on primary cardiac cells are scarce^33–35,43^, thus future experiments on primary cells are needed. When electroporating cells in parallel orientation with a 100 µs pulse, the pores start forming at approximately the same electric field independently of their elongation, while for perpendicular orientation, a higher electric field is needed for more elongated cells than for less elongated cells to achieve a similar level of electroporation. More experimental and numerical studies are needed to elucidate the mechanism for this observation.

### From single cell level to tissue level

A drawback of our study is that in *in vitro* experiments, several cells in a monolayer were exposed to electric pulses during a single experiment whereas modeling was done for a single cell. However, modeling several cells would significantly increase the computational cost and/or a coarser mesh would need to be used, all influencing the quality of the results and duration of the calculation. Indeed, agglomerates of cells impact the electric field distribution affecting the results obtained. It was shown that presence of other cells influences the induced transmembrane voltage^72,73^ and the permeability curve changes as higher electric fields are needed to electroporate the cells^74^. When working with cell cultures in *in vitro* experiments, however more cells allows for better statistics. Moreover, cells grow in close proximity to retain a specific cell phenotype. Our simplification of a single cell model is justified by the fact that, in tissues, similar responses were observed as on single cell level, with the exception of electric field which needs to be lower to achieve a similar effect in the case of tissues than with single cells^74^. In previous studies where the effect of cell orientation on electroporation was investigated, results similar to ours were obtained and reported on isolated cells^33,34^, on cell cultures^31^, *in vivo* in tissues^32^ and in modeling single cells^31^.

In tissues, however, the situation is different, as cells grow in three dimensions, there are different types of cells present, and general orientation of muscle fibers can vary within the same muscle, inter- as well as intraspecies. In the case of skeletal muscles, the orientation of muscles fibres is in general well established with the arrangement of muscle fibers relative to the axis of force generation. However, the structure of the heart is very complex and still under debate with respect to fibre orientations in the atrium ranging from −70° in the outer wall to +80° in the inner wall^75–77^. We believe that our *in vitro* study offers an important insight into achieving more homogeneous cell electroporation and achieving uniform lesions but at the same time points to new necessary *in vivo* experiments.

### Possible applications

Our experiments were performed on myocytes cell lines with the intent of treating heart arrhythmias with irreversible electroporation. For the second relevant clinical application, i.e., gene electrotransfer into skeletal muscles, experiments on skeletal muscles still need to be performed to confirm the universality of our finding. Gene electrotransfer was initially performed mainly with shorter pulses (<100 µs), but later it was switched to longer pulses (10–20 ms)^78^ and finally, to a combination of short high-voltage and long low-voltage pulses for electroporation and electrophoretic movement of DNA into the cell^8,79^. Different orientation of muscle fibers could be one of the reasons for not yet agreed best practices in gene electrotransfer. With longer pulses, the orientation of cells plays a significant role in the electroporation yield. Therefore, in electroporation of tissue with different cell orientation such as heart, where cell orientation changes from one location in the heart to another, as well as from epicardial to endocardial side of the wall^75–77^, or in fibers of skeletal muscles, which can be parallel, pennate, circular, convergent, rhomboid and deltoid, the orientation of electric field would need to be changed in order to electroporate all cells efficiently^80^. With pulses 1–10 µs long, the change of electric field direction would not be needed since cells parallel and perpendicular to the electric field are affected to a similar extent, which could lead to the homogeneous cell electroporation and uniform lesions.

Our study also opens new possibilities of pulse delivery protocols, which would electroporate elongated but differently oriented cells more homogeneously and uniformly. In addition to changing electric field orientation during the pulse delivery by replacing electrodes or switching between different electrodes, delivering short microsecond pulses where cell orientation has little effect on electroporation could be used. The use of pulses with the duration 1–10 µs that enable electroporation independent of cell orientation seems to be an elegant and efficient choice.

## Conclusions

When establishing pulse protocols for targeting elongated and differently oriented cells in electroporation-based treatments like cardiac ablation and DNA vaccination by muscle gene transfection, it should thus be taken into account that shorter pulses in the nanosecond range and longer pulses in the millisecond range may cause inhomogeneous and non-uniform electroporation, more pronounced than pulses of a few microseconds. Our experimental and numerical results show that nanosecond pulses caused higher electroporation of perpendicular cells whereas millisecond pulses led to a higher electroporation of parallel cells. In contrast, pulses of a few microseconds affected cells of both directions to the same extent. To achieve the most homogeneous and uniform effect, microsecond range pulses could thus be used in addition to changing electric field orientation.

## Materials and Methods

### Experimental part

#### Cells

H9c2 rat cardiac myoblast cell line (European Collection of Authenticated Cell Cultures ECACC 88092904, mycoplasma free) and AC16 human cardiomyocyte cell line (Merck Millipore, SCC109, mycoplasma free) were used. H9c2 cells were seeded in two well Lab-Tek chambered coverglass (Nunc, ThermoFisher Scientific, USA) one or two days before the experiments at 8 × 10^4^ and 5 × 10^4^ cells per well, respectively, in Dulbecco’s Modified Eagle Medium DMEM D6546 (Sigma-Aldrich, Germany) supplemented with 4 mM L-glutamine (Sigma), 10% FBS (Sigma) and antibiotics penicillin 1 U/ml (PAA, Austria), streptomycin 1 µg/ml (PAA) and gentamycin 50 µg/ml (Sigma) at 37 °C, 10% CO_2_ atmosphere in a humidified chamber.

AC16 cells were seeded in two well Lab-Tek chambered coverglass 1–3 days before the experiments at 13–2.5 × 10^4^ cells per well, respectively, in DMEM/F-12 D6434 (Sigma) supplemented with 2 mM L-glutamine (Sigma), 12.5% FBS (Sigma) and antibiotics penicillin 1 U/ml (PAA, Austria), streptomycin 1 µg/ml (PAA) at 37 °C, 5% CO_2_ atmosphere in a humidified chamber.

For nanosecond pulses, H9c2 cells were seeded in its culture medium on a coverglass (12 mm in diameter) in 24-well plates 1–2 days before the experiment at 5 × 10^4^ and 3 × 10^4^ cells per well, respectively. AC16 cells were seeded to 24-well plates 1–3 days before the experiment at 5–1.5 × 10^4^ cells per well, respectively.

#### Electroporation detection with Fura-2 AM

Electroporation was detected with a fluorescent calcium indicator Fura-2 acetoxymethyl ester (AM) (ThermoFisher Scientific). Fura-2 AM is a cell-permeant form of the dye that enters the cell and is cleaved by cell esterases into a cell-impermeant form. When cells are electroporated, calcium ions from the electroporation medium (DMEM, 1.8 mM Ca^2+^) enter cells and change the fluorescence spectrum of the dye that can be detected in ratiometric measurements^54,81^.

Cells were stained with 2 µM Fura-2 AM in their culture media (H9c2 in DMEM, AC16 in DMEM/F-12) at 37 °C for 30 min. After staining, cells were washed three times with fresh DMEM (H9c2 culture medium, for composition, see above). Then, fresh DMEM medium (H9c2 culture medium) was added, and electroporation was performed on both cell lines in the same medium. Cells were observed under an inverted epifluorescent microscope (Zeiss Axiovert 200) using 40× objective (LD Achroplan 40×/0.60 corr, Zeiss), Prime sCMOS camera (Photometrics, USA), and a heated microscope stage (37 °C), using a humidified CO_2_ chamber. Cells were illuminated with a monochromator (Polychrome) with Xe light at two excitation wavelengths (340 nm and 380 nm) and exposure time of 200 ms, using appropriate filter set (Chroma 71500: 400dclp BS 45° D510/40 m EM 0°, Chroma Technology Corporation, USA). Images were acquired with the software VisiView (Visitron, Germany) in a time-lapse acquisition mode (70 s, 15 images, one image every 5 seconds). Pulses were delivered at the 7th second of image acquisition. Background (no cells) was subtracted from the images. The ratio images were obtained by dividing the fluorescence image of cells excited at 340 nm with the image excited at 380 nm. Fura-2 340/380 ratio in all pixels was multiplied by a VisiView preset factor of 100 to add numerical precision to 16-bit images with integer pixel values. When internal calcium concentration increased due to calcium uptake, the Fura-2 340/380 ratio increased.

#### Exposure of cells to electric pulses

Cells were exposed to electric pulses using two different electrode configurations and four pulse generators. Electroporation with shorter pulse durations required higher electric field for the same effect as with longer pulse duration^54^. Therefore, we applied different electric field strengths for each of the pulse durations (Table 1). Electric field strengths were chosen experimentally according to fura response covering the whole range from no response, weak response, strong response up to the maximum response where cells were still capable of recovering (Supplement, Fig. S3).

For 100 ns pulses, Fura-2 labeled cells on round coverglass were placed on 40 µl drop of DMEM medium on top of gold micro-electrodes with a gap of 100 µm between the electrodes, 2.1 µm thick, mounted onto a cover glass^82^, with cells facing down (Fig. 6A). The coverglass was gently pushed down until the medium dislocated on top of the coverglass. The distance between coverglass with cells and the electrodes was determined using Prior ProScan III (Prior Scientific ltd., Cambridge, UK) and was between 30 and 50 µm, with an average distance of 40 µm. Pulses were delivered by the laboratory prototype (University of Ljubljana)^83^ Blumlein generator with 1 kV RF (radiofrequency) MOSFET switch (DE 475-102N20A, IXYS-RF, USA) and two 10 m, RG 58/U, 50 Ω transmission lines (Amphenol RF). Transmission lines were directly soldered to the microelectrodes, and 270 Ω resistor (TSF 270RJE, Ohmite) was used in parallel to the load to match the generator’s and load’s impedance. Voltage was measured on the electrode level by the oscilloscope (WaveSurfer 422, Teledyne LeCroy) using a high-voltage probe (PPE2KV, Teledyne LeCroy). Due to the positioning of cells on top of the electrodes, the exact electric field to which the cells were exposed was determined by numerical modeling of electric field distribution around the electrodes (Supplementary Fig. S1). For 100 ns pulse exposure, the microscope stage heating and CO_2_ chamber were not used to avoid the drying of the sample. Because of that, cells required a significantly longer period for recovery and were thus exposed to electric pulses only once: with the electric field set to 20, 26.6, 40 or 46.6 kV/cm.

For electroporation with 1 µs–10 ms, two parallel Pt/Ir wire electrodes, with 0.8 mm diameter and 4 mm distance between inner edges, were placed to the bottom of the Lab-Tek chamber (Fig. 6B). The electric field to which the cells were exposed was approximated as a ratio of voltage to distance between the inner edges^39^. Single pulses of 1 µs duration were delivered by the laboratory prototype pulse generator (University of Ljubljana) based on H-bridge digital amplifier with 1 kV RF MOSFETs (DE275-102N06A, IXYS, USA)^39,52^, of 10 – 1000 µs duration by the Electro cell B10 electroporator (BetaTech, France), and of 10 ms by the Intracell TSS20 Ovodyne generator (Abbotsbury Engineering Ltd, Saint Ives, UK). Voltage and current were measured by the oscilloscope (WaveSurfer 422) using a differential voltage probe (ADP305) and a current probe (CP030), all from LeCroy, USA (Fig. 6). The same cells were exposed to single pulses of the same duration but with increasing voltage. After each pulse exposure, cells were allowed to reseal 8 to 12 min, depending on the applied voltage, to restore low internal calcium concentration.

To test if the Fura-2 in our experiments is indeed an indicator of plasma membrane electroporation, i.e., an indicator of calcium uptake from the medium into the cell, we performed the following experiments. Cells dyed with Fura-2 were exposed to a single electric pulse of the same duration as before (1 µs – 10 ms) (Supplementary Fig. S2), at moderate electric field strengths, but in calcium-depleted medium – SMEM medium without Ca^2+^ (Minimum Essential Medium Eagle, Spinner Modification, Sigma Merck M8167) but with 5 μM EGTA (ethylene glycol-bis(β-aminoethyl ether)-N,N,N′,N′-tetraacetic acid). The fluorescence ratio did not change after applying an electric pulse of 1 µs – 10 ms duration (Supplementary Fig. S2A). After 12 min, the medium was changed to DMEM medium (contains 1.8 mM calcium) and 13–15 min after the first pulse, cells were exposed to the same pulse again. This time, the same cells responded with a rise in Fura-2 340/380 ratio. Thus, in our experiments, we detect uptake of calcium ions from the medium not from the internal stores. Different concentrations of phenol red in media and residual EGTA in changed medium may contribute to different baselines of Fura-2 340/380 ratio. However, for 100 ns pulses, changing the medium was not possible due to the setup configuration (cells on coverglass on top of the electrodes). Therefore, we exposed the cells to 100 ns electric pulses only in SMEM without Ca^2+^ but with EGTA and compared these results to the ones performed in DMEM medium (Supplementary Fig. S2B). In conditions without Ca^2+^ but with EGTA, the internal Ca^2+^ concentration slightly rises but not to the same amount as in conditions with Ca^2+^ present. This means that some of Ca^2+^ ions are released from internal stores but the majority is taken up from extracellular medium^84^ and can, therefore, be a method for detecting plasma membrane electroporation.

#### Image analysis

Image analysis was done using an open-source image-processing program ImageJ (National Institutes of Health, Bethesda, MD). Cells in ratio images were manually encircled and mean fluorescence intensity ratio Fura-2 340/380 was determined for each cell. At each experiment, 4–30 cells were analyzed, and the average ratio Fura-2 340/380 at 8 s after the pulse application (the peak calcium concentration, see Supplementary Fig. S3) was determined for all experiments. The orientation of cells in all experiments including controls was also determined with the use of ImageJ. Cells that were previously encircled were fitted with an ellipse. For parallel and perpendicular cells, two conditions had to be met. First, the longer axis (a) was at least twice as long as the shorter axis (b): a > 2b. This means that the cells were substantially elongated and not round. Second, their longer axis (a) was parallel or perpendicular to the electric field. Since cells that are 100% parallel or perpendicular are very rare, we determined the boundaries for each orientation to be 0° ± 20° from the main axis for the parallel and 90° ± 20° for the perpendicular. Cells that did not meet these conditions were treated as “the rest”, i.e., being essentially round and/or not oriented parallel or perpendicular to the electric field. In each experiment, at least one cell fit in each group (parallel, perpendicular, the rest). The average Fura-2 340/380 ratio of parallel, perpendicular, the rest and all cells was calculated for each experiment using Excel (Microsoft Corp., Redmond, WA) and baseline (at time zero) was subtracted from the results. For each experiment, the difference in Fura-2 340/380 ratio (baseline subtracted) between parallel and perpendicular cells (parallel-perpendicular) was calculated and statistically analyzed.

In AC16 cells, at higher electric field strengths cells started to round into a spherical shape (Supplementary Fig. S4). This way, cells lost their elongated form and could not be treated as parallel/perpendicular anymore. Therefore, AC16 were only analyzed at electric field strengths where rounding was not yet observed (lower than H9c2, where rounding was not observed even at the highest tested electric field strengths).

### Statistical analysis

Statistical analysis was performed using Excel and SigmaPlot 11.0 (Systat Software, Chicago, IL). The results in figures and the text are expressed as median Fura-2 340/380 ratio difference between parallel and perpendicular cells, and statistically significant differences (p < 0.05) were determined by the Kruskal-Wallis One Way Analysis of Variance on Ranks (our data did not follow the normal distributions), followed by Multiple Comparisons versus Control Group (the Dunn’s Method). Normality was tested with the Shapiro-Wilk test. The results of statistical analyses are shown in the Appendix, Tables A1a and A1b. When the difference parallel-perpendicular is negative, it means that the perpendicular cells are electroporated more than the parallel, and when the difference is positive, it means that the parallel cells are electroporated more than the perpendicular.

### Modeling part

The model was constructed as in^44,85^ where pore formation was modeled with the asymptotic pore equation^86^ in Comsol Multiphysics (v5.3a, www.comsol.com, Stockholm, Sweden). In brief, a cell was shaped as an elongated spheroid with the length (diameter) of the two short axes and one long axis d_long_ = 90 µm and d_short_ = d_long_/2 (ratio 1:2, the minimum ratio for which cells were treated as a spheroid in experiments, Fig. 7A) or d_short_ = d_long_/4 (ratio 1:4, Fig. 7B). The size was determined as a representative size of the cells in the experiments. The average length of cells in the experiments was 90 µm and 70 µm for the long axis and the ratio of the length of the long axis vs the short axis of 3.7 ± 2.0 and 3.14 ± 0.97, for H9c2 and AC16, respectively.

Due to the attachment of cells to the bottom of the dish in the *in vitro* experiments, cells were more half-spheroids (Fig. 7C) than whole spheroids (Fig. 7A,B). However, in the experiments, the bottom of the dish was electrically insulated, and from an electrical point of view, the problem was symmetrical and numerical calculations are equivalent for both cases. Also, due to the assumed symmetry of the cell in *x*- and *y*-axis, we could model only 1/8 of the cell and significantly decrease the computational cost. The size of the simulation cube was 400 × 400 × 400 µm. We confirmed that the box size did not affect the results. Mesh was refined until there was a negligible effect of the mesh on the results. The 3-dimensional simulation was made in AC/DC module, electric currents physics, study in the time domain. Voltage was applied as a trapezoidal pulse to two opposite boundaries of the box, either parallel to the long axis of the spheroid, or perpendicular to it. The pulse had to be trapezoidal due to convergence problems with a perfectly square pulse. Other boundaries of the box were modeled as electrically insulated. Cell membrane was modeled as a boundary condition contact impedance^87^:

1 $$n\cdot {J}_{1}=\frac{1}{{d}_{m}}({\sigma }_{m}+{\varepsilon }_{0}{\varepsilon }_{m}\frac{\partial }{\partial t})({V}_{1}-{V}_{2}),$$

2 $$n\cdot {J}_{2}=\frac{1}{{d}_{m}}({\sigma }_{m}+{\varepsilon }_{0}{\varepsilon }_{m}\frac{\partial }{\partial t})({V}_{2}-{V}_{1}),$$

where *n* is the normal vector, *J* is the electric current density, *V*_1_ and *V*_2_ are potentials on both sides of the membrane, is the permittivity of the vacuum, *d*_*m*_ is cell membrane thickness, *ε*_*m*_ is permittivity of the cell membrane (Table 2) and ε_0_ is the permittivity of the vacuum. The asymptotic pore equation was calculated as a weak form boundary partial differential equation:

3 $$\frac{dN}{dt}=\alpha \,\exp \left({\left(\frac{{U}_{m}}{{V}_{ep}}\right)}^{2}\right)\left(1-\frac{N}{{N}_{0}}\exp {(-q\left(\frac{{U}_{m}}{{V}_{ep}}\right)}^{2}\right),$$

**Table 2:** Parameters, used in the numerical study, their values and references.

Parameter	Symbol	Value
Relative dielectric permittivity of the intracellular space	*ε*_*i*_	80 (−)^55^
Relative dielectric permittivity of the extracellular space	*ε*_*e*_	80 (−)^55^
Relative dielectric permittivity of the cell membrane	*ε*_*m*_	5 (−)^55^
Electric conductivity of the intracellular space	*σ*_*i*_	0.3 S/m^55^
Electric conductivity of the extracellular space	*σ*_*e*_	1 S/m [typical value for a high conductive electroporation buffer]
Electric conductivity of the cell membrane	*σ*_*m0*_	0.3 µS/m^55^
Pore radius	*r*_*p*_	0.76 nm^44^
Electroporation parameter	*α*	10^9^ m^−2^s^−1^ ^44^
Characteristic voltage of electroporation	*V*_*ep*_	0.258 V^44^
Equilibrium pore density when V_m_ = 0 mV	*N*_*0*_	1.5 × 10^9^ m^−2^ ^86^
Electroporation constant	*q*	1.46 (−)^44^
Membrane thickness	*d*_*m*_	10 nm^88^
Electric conductivity of a pore	*σ*_*p*_	(*σ*_*e*_- *σ*_*i*_)/ln(*σ*_*e*_- *σ*_*i*_)^44^

With *N* being the pore density, *U*_*m*_ the transmembrane voltage and other parameters are explained in Table 2. The initial value of *N* was *N*_0_ as the resting voltage was modeled to be 0 mV^86^.

The conductivity of the cell membrane (*σ*_*m*_) was a function of the pore density and was calculated as:

4 $${\sigma }_{m}={\sigma }_{m0}+N\frac{2\pi {{r}_{p}}^{2}{\sigma }_{p}{d}_{m}}{\pi {r}_{p}+2{d}_{m}},$$

Where *σ*_*m*0_ is the electric conductivity of the non-electroporated membrane, *r*_*p*_ is pore radius (Table 2). In the parametric study, we varied pulse duration and applied an electric field, scaled according to the pulse duration (Fig. 8). Scaling was achieved by using equations from^54^, where the electric field was deemed as equivalent, if 70% cells were fura positive. One trapezoidal pulse of different amplitudes was applied either perpendicularly or in parallel to the long axis of a cell. The rise-time and fall-times of the pulses were one-hundredth of a pulse length. Shorter pulses must be of a higher electric field to achieve the same effect, which is described by a strength-duration curve^52,54^. We tested 1) the hyperbolic relation of the strength-duration curve:

5 $$E=520.9\left[\frac{V}{cm}\right]+1.43\left[\frac{V}{cm}\right]\frac{1}{{t}_{pulse}}$$

where *t*_*pulse*_ is the duration of the pulse in milliseconds and 2) the Saulis pore equation fitted on the experimental results of electroporation detected by calcium ions^54^. As a result, we integrated the pore density over 1/8 of the cell membrane (modeled part of the membrane) at the end of the pulse to obtain pore number. We also tested another approach and considered the area as electroporated if the pore density was above 10^13^ pores/m^2^, compared the fraction of electroporated areas and obtained similar results (Supplementary Fig. S7).

**Table A1:** Statistical parameters of difference in the Fura-2 340/380 ratio between parallel and perpendicular cells (Fig. 2B,C: Median, Q1 and Q3 (A.U.), n – number of repetitions).

Pulse duration	Electric field strength	Median (A.U.)	Q1	Q3	n	H	Degrees of freedom	P	p<0.05
**a: H9c2 cells**									
100 ns						22.5	4	<0.001	
	0 kV/cm	0.09	−0.12	0.34	25				
	20.0 kV/cm	−5.82	−15.36	−4.25	6				*
	26.6 kV/cm	−6.10	−14.18	−1.45	9				
	40.0 kV/cm	−7.58	−15.63	−5.67	5				*
	46.6 kV/cm	−10.30	−13.40	−2.40	5				
1 µs						0.8	5	0.976	
	0 V/cm	−0.10	−0.45	0.35	3				
	1000 V/cm	0.15	−0.83	0.18	3				
	1400 V/cm	0.25	−6.36	0.58	3				
	1800 V/cm	−0.47	−4.52	1.09	3				
	2200 V/cm	−1.15	−4.31	0.96	3				
	2500 V/cm	−0.60	−5.25	0.34	3				
10 µs						5.5	6	0.487	
	0 V/cm	−0.40	−0.41	−0.23	3				
	400 V/cm	0.63	0.20	0.86	3				
	600 V/cm	1.07	−0.32	3.60	3				
	800 V/cm	−2.16	−3.10	−0.15	3				
	1000 V/cm	0.78	−4.98	0.91	3				
	1200 V/cm	0.66	−1.92	1.25	3				
	1400 V/cm	2.16	−5.76	2.33	3				
100 µs						0.7	4	0.951	
	0 V/cm	0.02	−0.06	0.37	3				
	200 V/cm	1.25	−0.38	4.91	3				
	300 V/cm	1.00	−2.10	2.53	3				
	400 V/cm	0.18	−0.48	2.52	3				
	500 V/cm	0.11	−0.52	11.36	3				
1 ms						16.7	4	0.002	
	0 V/cm	−0.01	−0.14	0.29	4				
	125 V/cm	7.72	7.10	9.19	4				
	200 V/cm	13.07	12.07	14.58	4				*
	300 V/cm	6.50	3.91	9.13	4				
	400 V/cm	0.06	−3.91	1.23	4				
10 ms						23.3	6	<0.001	
	0 V/cm	0.18	−0.69	0.55	5				
	50 V/cm	0.60	−0.76	1.59	5				
	75 V/cm	3.85	1.70	5.01	5				
	100 V/cm	7.32	5.88	9.24	5				*
	125 V/cm	9.14	5.75	12.15	5				*
	150 V/cm	7.62	3.84	10.12	5				*
	175 V/cm	4.88	2.22	6.82	5				
**b: AC16 cells**									
100 ns						27.1	4	<0.001	
	0 kV/cm	−0.08	−0.30	0.05	25				
	20.0 kV/cm	−7.02	−13.96	−2.47	7				*
	26.6 kV/cm	−6.33	−9.39	−1.20	7				
	40.0 kV/cm	−10.93	−18.26	−7.72	5				*
	46.6 kV/cm	−13.99	−25.73	−5.47	6				*
1 µs						7.5	5	0.184	
	0 V/cm	0.07	0.07	0.09	3				
	1000 V/cm	0.14	0.02	0.64	3				
	1400 V/cm	−0.90	−0.91	0.47	3				
	1800 V/cm	−1.27	−4.82	1.03	3				
	2200 V/cm	−2.83	−8.61	0.19	3				
	2500 V/cm	−7.37	−8.94	−3.09	3				
10 µs						10.8	5	0.056	
	0 V/cm	−0.01	−0.22	0.08	3				
	400 V/cm	0.72	−0.34	1.03	3				
	600 V/cm	2.19	−3.04	2.56	3				
	800 V/cm	4.11	−6.73	4.18	3				
	1000 V/cm	−5.18	−11.89	−4.86	3				
	1200 V/cm	−11.31	−17.56	−10.29	3				
100 µs						5.5	4	0.237	
	0 V/cm	0.09	−0.07	0.46	3				
	200 V/cm	4.27	3.49	5.94	3				
	300 V/cm	7.62	3.06	12.91	3				
	400 V/cm	3.44	−6.57	10.07	3				
	500 V/cm	−5.62	−7.77	4.29	3				
1 ms						6.5	2	0.011	
	0 V/cm	0.01	−0.13	0.08	3				
	125 V/cm	2.46	0.06	5.87	3				
	200 V/cm	18.81	8.55	19.03	3				*

Statistical analysis by the Kruskal- Wallis One Way Analysis of Variance on Ranks (H, degrees of freedom, P) followed by Multiple Comparisons versus Control Group (Dunn’s Method, *p < 0.05). Table A1a: H9c2 cells, Table A1b: AC16 cells.

## Supplementary information

Supplemental information.

## Data Availability

All data generated or analysed during this study are included in this published article (and its supplementary information files).
