# Supplementary material for: Short microsecond pulses achieve homogeneous electroporation of elongated biological cells irrespective of their orientation in electric field
Source: Sci Rep. 2020 Jun 4;10:9149. doi: 10.1038/s41598-020-65830-3 (PMC7272635; doi:10.1038/s41598-020-65830-3)
Supplement: Supplementary file 1 — Supplemental information. [file 41598_2020_65830_MOESM1_ESM.docx]

# Supplementary file - Experimental and numerical evaluation of electroporation of cardiomyocytes with pulses of a nanosecond to millisecond duration

Janja Dermol-Černe, Tina Batista Napotnik, Matej Reberšek, Damijan Miklavčič*

University of Ljubljana, Faculty of Electrical Engineering, Tržaška cesta 25, 1000 Ljubljana, Slovenia

Figure S1: Electric field distribution in kV/cm when 600 V are applied to the electrode setup, used for nanosecond pulse application. The model was constructed in Comsol Multiphysics V5.3 (Comsol, Sweden) as a stationary study using the electric currents module. One electrode was set to $V_{1} = 300V$ and another to $V_{2} = -300V$ to obtain the potential difference of 600 V and avoid the numerical problems with voltages around 0. In our model, cells were exposed to the electric field 40 µm above the electrode plane where the electric field is approximately homogeneous and around 26.6 kV/cm.


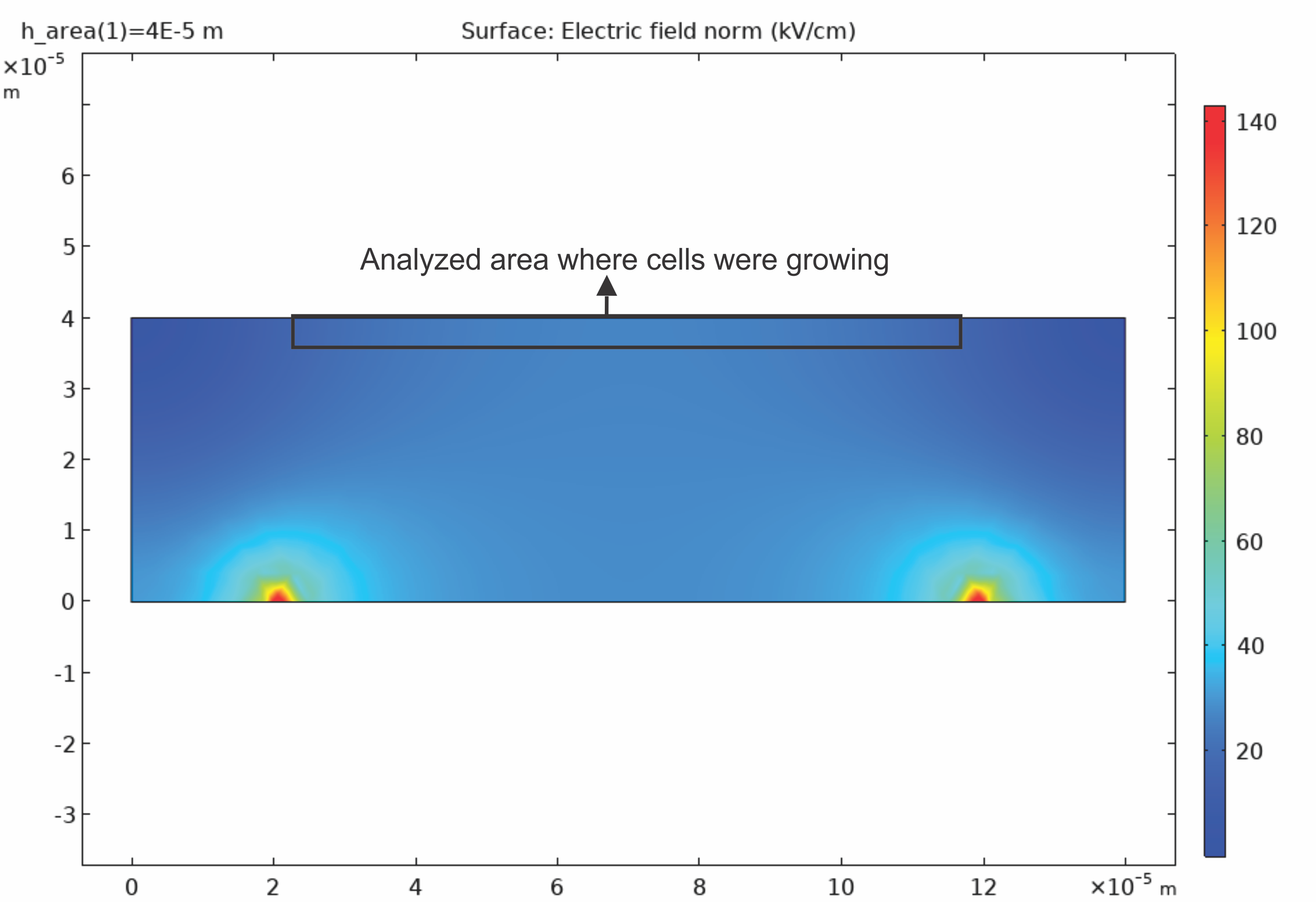


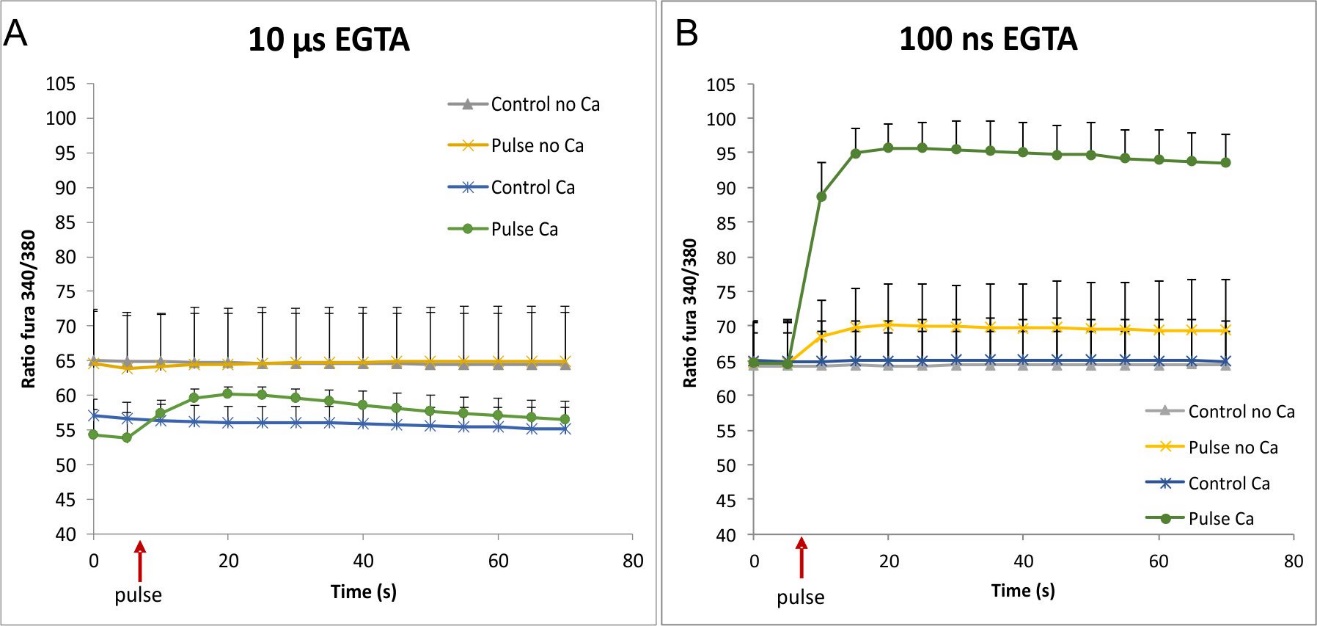
Figure S2: Experimental results of electroporation of cardiomyocytes H9c2 in conditions with or without external calcium: Fura-2 ratio 340/380 over time. Experimental electroporation of H9c2 cells of different orientations was monitored by Ca^2+^ uptake with a fluorescent calcium indicator Fura-2, image acquisition was done every 5 s. A) The same cells were exposed to a single pulse of 10 μs, 800 V/cm, 7 s after image acquisition start (noted with red arrow), first in conditions without external Ca^2+^ but with EGTA present, and after that, the medium was changed to DMEM culture medium with Ca^2+^ present. Results are presented as average from three experiments (12-20 cells per experiment analyzed). Similar results were obtained using 1 μs – 10 ms pulses. B) H9c2 cells were exposed to a single pulse of 100 ns, 40 kV/cm in conditions either without external Ca^2+^ but with EGTA present or in DMEM culture medium with Ca^2+^ present. Results are presented as average from six experiments for no Ca^2+^ conditions (5-13 cells per experiment analyzed) and from five experiments for DMEM conditions (5-14 cells per experiment analyzed), vertical bars represent SD. Controls are experiments without pulse application.


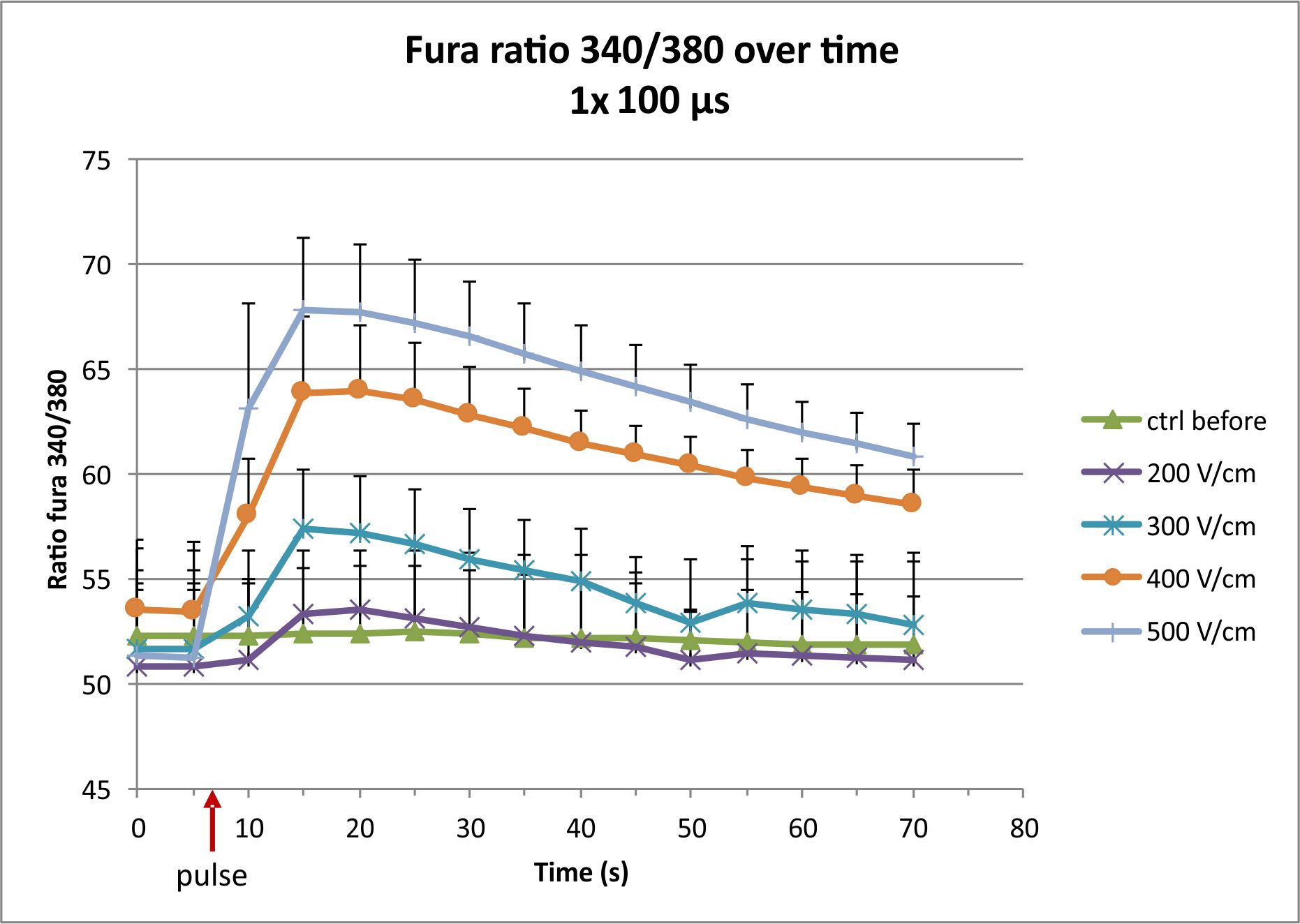


Fig. S3: Experimental results of electroporation of cardiomyocytes H9c2: Fura-2 ratio 340/380 over time. Experimental electroporation of H9c2 cells of different orientations was monitored by Ca^2+^ uptake with a fluorescent calcium indicator Fura-2, image acquisition was done every 5 s. The same cells were exposed to single pulses of 100 μs duration but with increasing voltage (200 – 500 V/cm) 7 s after image acquisition start (noted with red arrow). After each pulse exposure, cells were allowed to recover. Results are presented as average from three experiments (13-21 cells per experiment analyzed), vertical bars represent SD.


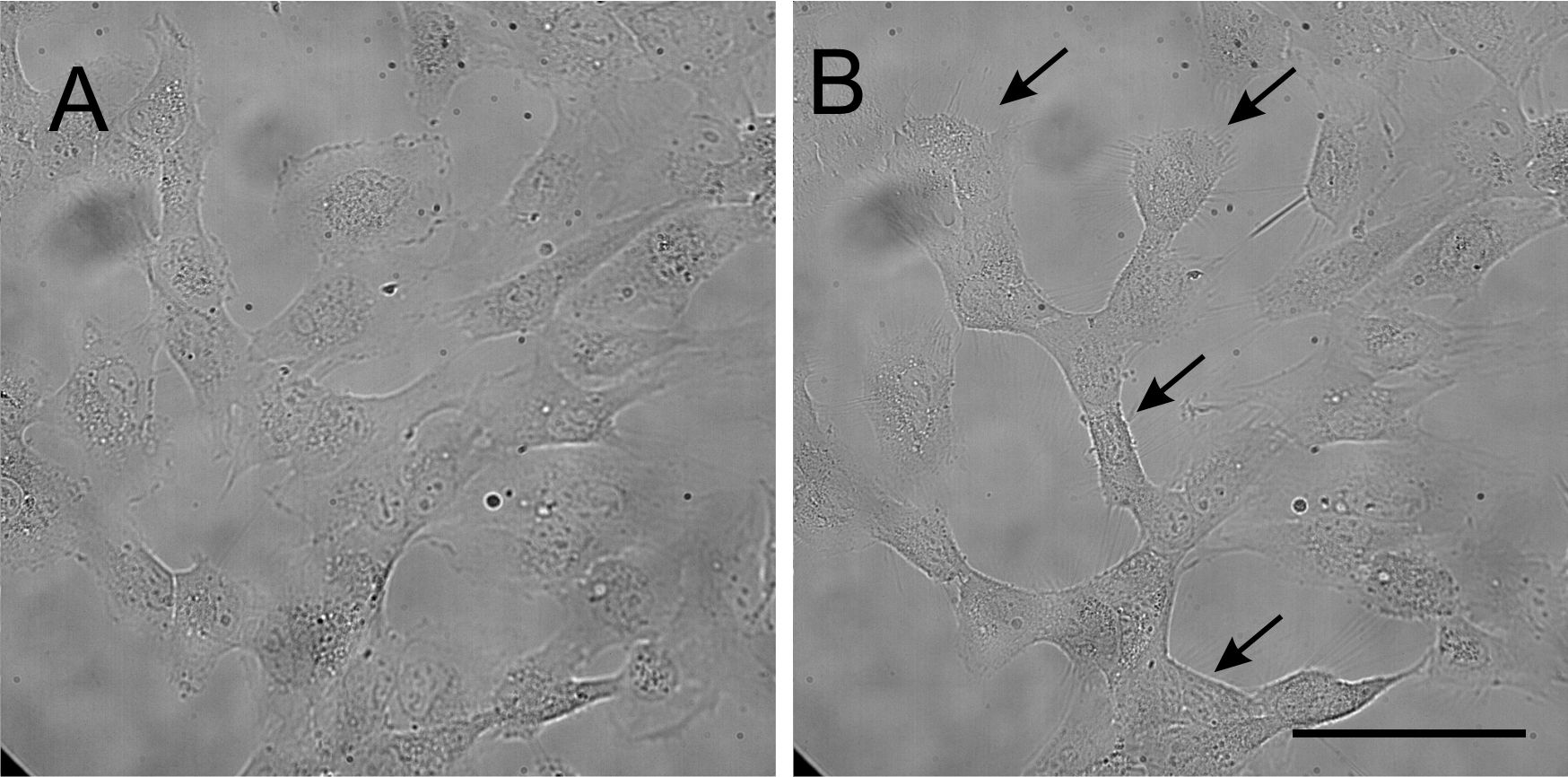


Fig. S4: Image of AC16 cells before (A) and after electroporation (B). In S2B, cells were exposed to subsequent single pulses of 1 ms with increasing voltages (125, 200, 300, 400 V/cm), with recovery time in between. Arrows point to rounded cells. Scalebar: 100 μm.

# Modeling

A B


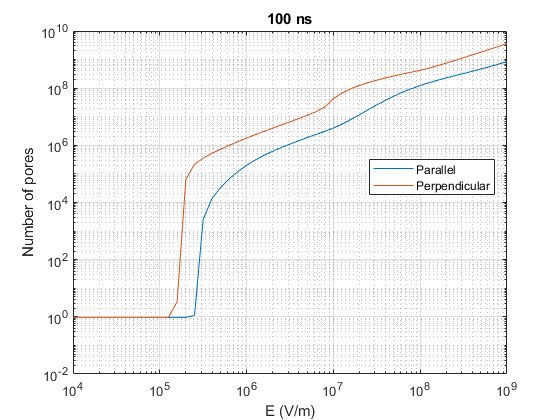

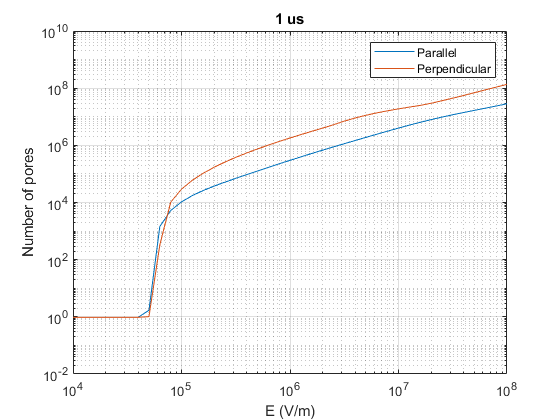


C


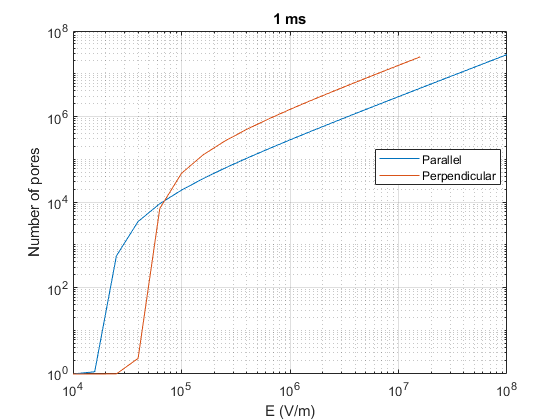


Figure S5: Numerically determined number of pores on cell membrane as a function of electric field when one pulse of different duration A) 100 ns, B) 1 µs and C) 1 ms is applied.


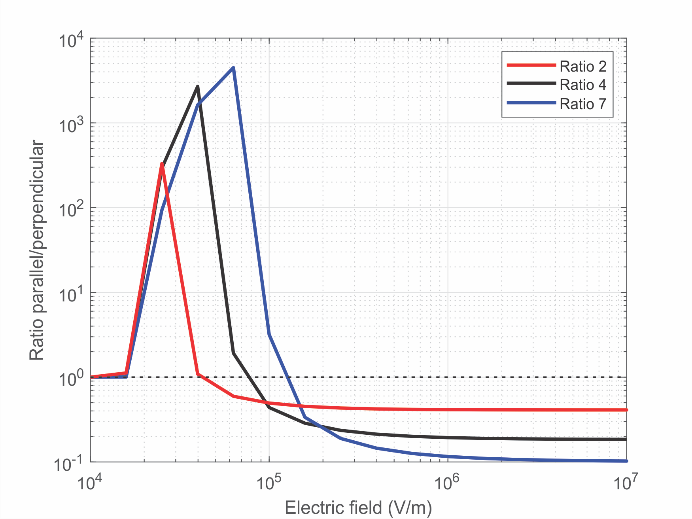


Fig. S6: The ratio of formed pores for parallel vs perpendicular cell orientation as a function of applied electric field when one 100 µs long pulse is applied. Three different cell geometries are shown – less elongated cells (ratio long to short axis 2, red line), the elongation observed in our experiments (ratio 4, black line) and the elongation in primary cells (ratio 7). The ratio parallel/perpendicular around 1 means that both cell orientations were similarly effective. The width of the curve above 1 (dashed line) indicates where electroporation is inhomogeneous, *i.e.* cell orientation affects electroporation. The width is larger for larger ratios, thus, for larger ratios, homogeneous electroporation of cells oriented differently is more difficult to achieve than for smaller ratios.


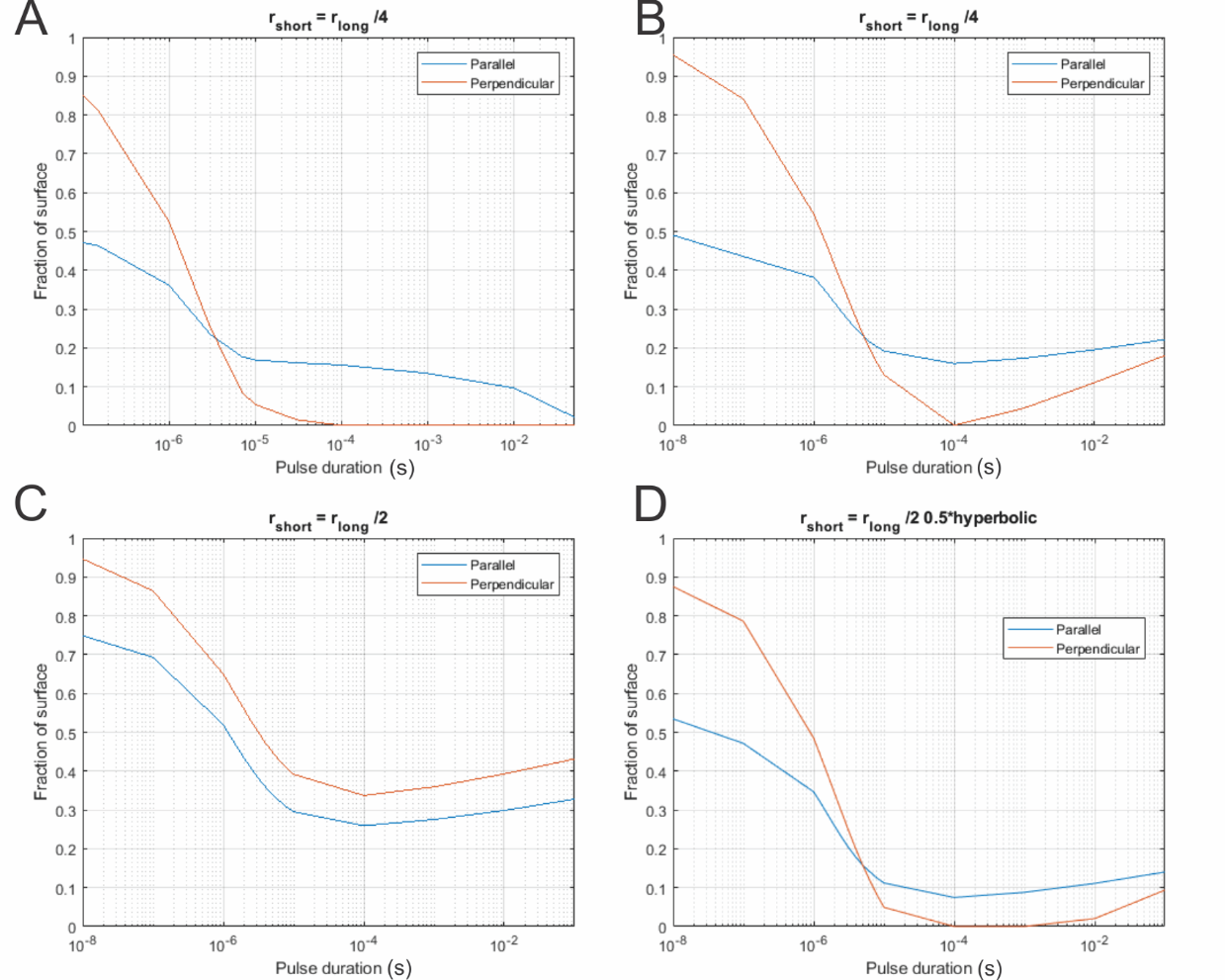


Figure S7: Fraction of the electroporated area as a function of pulse duration when pulses of equivalent amplitude are applied for two different cell geometries. A) When short axis is one quarter of the length of the long axis, and equivalent parameters are obtained with the hyperbolic equation (crossover at 6 µs) or B) with the Saulis pore equation (crossover at 3 µs) or c) when short axis is one half of the length of the long axis, and equivalent parameters are obtained with the hyperbolic equation (no crossover observed) or D) hyperbolic equation, scaled for a factor of 0.5 to take into account larger cell geometry (crossover at 4 µs).
